# Association Mapping Analysis of Fatty Acid Content in Different Ecotypic Rapeseed Using mrMLM

**DOI:** 10.3389/fpls.2018.01872

**Published:** 2019-01-04

**Authors:** Mingwei Guan, Xiaohu Huang, Zhongchun Xiao, Ledong Jia, Shuxian Wang, Meichen Zhu, Cailin Qiao, Lijuan Wei, Xinfu Xu, Ying Liang, Rui Wang, Kun Lu, Jiana Li, Cunmin Qu

**Affiliations:** ^1^Chongqing Engineering Research Center for Rapeseed, College of Agronomy and Biotechnology, Southwest University, Chongqing, China; ^2^Academy of Agricultural Sciences, Southwest University, Chongqing, China

**Keywords:** *Brassica napus* L., candidate genes, GWAS, mrMLM, fatty acid content

## Abstract

*Brassica napus* L. is a widely cultivated oil crop and provides important resources of edible vegetable oil, and its quality is determined by fatty acid composition and content. To explain the genetic basis and identify more minor loci for fatty acid content, the multi-locus random-SNP-effect mixed linear model (mrMLM) was used to identify genomic regions associated with fatty acid content in a genetically diverse population of 435 rapeseed accessions, including 77 winter-type, 55 spring-type, and 303 semi-winter-type accessions grown in different environments. A total of 149 quantitative trait nucleotides (QTNs) were found to be associated with fatty acid content and composition, including 34 QTNs that overlapped with the previously reported loci, and 115 novel QTNs. Of these, 35 novel QTNs, located on chromosome A01, A02, A03, A05, A06, A09, A10, and C02, respectively, were repeatedly detected across different environments. Subsequently, we annotated 95 putative candidate genes by BlastP analysis using sequences from *Arabidopsis thaliana* homologs of the identified regions. The candidate genes included 34 environmentally-insensitive genes (e.g., *CER4, DGK2, KCS17, KCS18, MYB4*, and *TT16*) and 61 environment-sensitive genes (e.g., *FAB1, FAD6, FAD7, KCR1, KCS9, KCS12*, and *TT1*) as well as genes invloved in the fatty acid biosynthesis. Among these, *BnaA08g08280D* and *BnaC03g60080D* differed in genomic sequence between the high- and low-oleic acid lines, and might thus be the novel alleles regulating oleic acid content. Furthermore, RT-qPCR analysis of these genes showed differential expression levels during seed development. Our results highlight the practical and scientific value of mrMLM or QTN detection and the accuracy of linking specific QTNs to fatty acid content, and suggest a useful strategy to improve the fatty acid content of *B. napus* seeds by molecular marker-assisted breeding.

## Introduction

Rapeseed (*Brassica napus* L.) is one of the most important oil crops in the world, providing not only edible vegetable oil but also its potential use in lubricants and biofuels (Saeidnia and Gohari, 2012). However, the physical, chemical, and nutritional qualities of rapeseed oil depend mainly on its fatty acid composition, which consists approximately of 60% oleic acid (C18:1), 4% palmitic acid (16:0), and 2% stearic acid (18:0) (Bauer et al., 2015; Wen et al., 2015). Rapeseed oil is considered by many nutritionists to be ideal for human nutrition and superior to many other plant oils (Zhao et al., 2008; Qu et al., 2017), as it can be heated to high temperatures without smoking (Miller et al., 1987), and reduces levels of undesirable low-density lipoprotein cholesterol in the blood plasma, reducing the risk of arteriosclerosis (Chang and Huang, 1998). Optimizing the fatty acid composition is an important breeding objective for rapeseed cultivar development.

In *B. napus*, fatty acid metabolism is influenced by both genotype and environmental factors. Efforts to improve the oil quality have yielded many high oleic acid *Brassica* lines, including *B. rapa* (Tanhuanpää et al., 1996), *B. carinata* (Velasco et al., 1997), and *B. napus* (Pleines and Friedt, 1989; Fei et al., 2012). Further, oleic acid concentrations >70% have already been achieved in rapeseed through hybrid breeding methods (Zhang et al., 2009). Fatty acid content is a typical quantitative trait controlled by multiple genes that regulate its desaturation (Wang et al., 2015; Chen et al., 2018), and numerous quantitative trait loci (QTLs) for fatty acids have been mapped to all 19 chromosomes of *B. napus*, with most being found on chromosomes A01, A02, A03, A08, A10, C03, A04, A07, A09, C01, C06, and C08 (Burns et al., 2003; Zhao et al., 2008; Liu and Li, 2014; Bauer et al., 2015; Lee et al., 2015; Teh, 2015; Wen et al., 2015; Javed et al., 2016). With the increasing availability of whole-genome-sequences and SNP array development, association mapping represents a powerful approach for dissecting the genetic basis of complex quantitative traits at high resolution, which could significantly increase the precision of estimating QTL locations (Meuwissen and Goddard, 2000). Recently, genome-wide association studies (GWAS) have been performed to detect the genetic variation associated with important agronomic traits in rapeseed using the Illumina Infinium *Brassica* 60K SNP array (Delourme et al., 2013; Li et al., 2014; Lu et al., 2014; Hatzig et al., 2015; Luo et al., 2015), including seed weight and quality (Li et al., 2014), seed oil content in a panel of 521 rapeseed accessions (Liu et al., 2016), and the composition of seven fatty acids (Qu et al., 2017). Although these studies have revealed loci for associated with fatty acid traits, no beneficial alleles have been detected within the *B*. *napus* accessions.

Numerous studies showed that *FATTY ACID DESATURASE 2* (*FAD2*) is the major gene responsible for the desaturation of oleic acid to linolenic acid (Hu et al., 2006; Peng et al., 2010; Yang et al., 2012), and four paralogs of *FAD2* were previously identified in *B. napus* (Scheffler et al., 1997; Yang et al., 2012). These paralogs were mainly expressed in the developing seeds, suggesting possible roles in controlling oleic acid content in *B. napus* (Xiao et al., 2008). In addition, *KCS18*, is known to play a crucial role in regulating erucic acid biosynthesis in *B. napus* (Wang et al., 2008; Wu et al., 2008; Li et al., 2014). However, the identified QTL were not cloned and undertaken for contributing to the minor fatty acids. Furthermore, the genetic basis of fatty acid synthesis is still unclear.

The multi-locus random-SNP-effect mixed linear model (mrMLM) is emerged as a powerful tool for quantitative trait nucleotide (QTN) detection and QTN effect estimation for complex traits (Wang et al., 2016; Li et al., 2017; Chang et al., 2018; Peng et al., 2018). For example, Li et al. (2017) detected 38 significantly-associated loci and identified numerous highly-promising candidate genes (e.g., *TAC1, SGR1, SGR3*, and *SGR5*), for branch angle across 472 rapeseed accessions. Zhang et al. (2018) identified 127 significant QTNs for stalk lodging resistance-related traits using mrMLM in a population of 257 maize inbred lines. As reported by Ma et al. (2018), 127 significant QTNs with maize embryonic callus regenerative capacity were identified in a population of 144 maize inbred lines, and many candidate genes were reported to relate with auxin transport, cell fate, seed germination, or embryo development, respectively. In the present study, we analyzed the fatty acid composition in 77 winter varieties, 55 spring varieties, and 303 semi-winter varieties of rapeseed grown in three environments, and genotyped all of the accessions using the high-through *Brassica* 60K SNP array (Clarke et al., 2016). Then, 32,543 SNPs from the 60K SNP array were used for genome-wide association analysis usingmrMLM. In total, 149 QTNs were identified using mrMLM, suggesting that this is an effective model for identifying candidate genes underlying complex traits. Subsequently, 95 candidate genes were annotated using BlastP against *A. thaliana* homologs, providing insight into the genetic control of fatty acid content in *B. napus*. Furthermore, novel fatty acid content-associated SNPs identified here may be useful for marker-based breeding programs aimed at improving the fatty acid content of *B. napus* seeds.

## Materials and Methods

### Plant Materials

A diversity panel consisting of 55 spring, 77 winter, and 303 semi-winter rapeseed accessions (*B. napus*; Supplementary Table S1) was used for the association analysis. These accessions were grown in three growing seasons (2015–2016, 2016–2017, and 2017–2018) in Beibei (106.38°E, 29.84°N), Chongqing, China. Three rows of 10–12 plants per accession were established in the experimental fields with a randomized complete block design and three replications. Self-pollinated seeds were harvested from plants at complete physiological maturity and used for the fatty acid analysis.

### Fatty Acid Measurement and Statistical Analysis

Seeds (200 mg) were homogenized with a pestle and extracted in 2 mL petroleum ether:ether (1:1) for 40 min, and methylated with 1 mL KOH/methanol (0.4 mol L^−1^). The supernatants separated by adding distilled water were identified by gas-liquid chromatography on a Model GC-2010 (Shimadzu, Kyoto, Japan). Chromatographic analysis was carried out using a fused silica capillary column DB-WAX (30 m × 0.246 mm × 0.25 um) with default parameters (Qu et al., 2017). Fatty acid profiles were calculated as a percentage of total fatty acids in the seeds, and optimized with an R script of the best linear unbiased prediction (BLUP) (Merk et al., 2012). The resulting values for each accession were used in the association analysis. All experiments were performed in triplicate, and the mean, standard deviation (SD), coefficient of variation (CV), and minimum (Min) and maximum (Max) values of the oleic acid content were calculated using SPSS 15.0 (IBM Corp, Armonk, NJ, USA).

### SNP Genotyping Data Acquisition and Analysis

The methods used for SNP genotyping and mapping were described in previous reports of Li et al. (2014) and Liu et al. (2016). Using the *Brassica* 60K Illumina® Infinium SNP array (Clarke et al., 2016), the genotype of each accession was generated at the National Subcenter of Rapeseed Improvement in Wuhan (Huazhong Agricultural University, Wuhan, China). The low quality SNPs (call frequency < 0.9 and a minor allele frequency ≤ 0.05) were filtered in all accessions. In addition, SNPs not accurately mapped to the *B. napus* genome were excluded. The probe sequences of 52,157 SNPs were used to perform a local BlastN search against the *B. napus* “Darmor-*bzh*” reference genome (version 4.1, http://www.genoscope.cns.fr/brassicanapus/data/; Chalhoub et al., 2014) using our previously published method (Wei et al., 2015). In total, 32,543 SNPs were analyzed further.

The Q matrix of population structure was calculated by a Bayesian model-based analysis in STRUCTURE 2.1 (Pritchard et al., 2000) with published parameters of Falush et al. (2003) and Qu et al. (2017). The optimal number of *K* values (*K* = 2; Supplementary Figure S1) was determined using the Evanno method (Evanno et al., 2005). The Q matrix was selected as the fixed covariate in the subsequent association analysis (Gajardo et al., 2015). To visualize genetic relatedness among all genotypes, the principal component analysis (PCA) was constructed using the GCTA tool (Yang et al., 2011). The relative kinship matrix for each association panel was calculated using SPAGeDi (Hardy and Vekemans, 2002), and the negative values were defined as zero between two individuals, following the method of Yu et al. (2006).

### Genome-Wide Association Analysis

The mrMLM significantly improved the power and precision of the GWAS, which was previously used in *B. napus* (Li et al., 2017). Therefore, the multi-locus GWAS method (mrMLM, https://cran.r-project.org/web/packages/mrMLM.GUI/index.html) was performed to evaluate the trait-SNP association analysis in this study (Wang et al., 2016). Moreover, the phenotypic and genotypic datasets, kinship (K), and population structure (Q) were imported into the R package mrMLM, and significantly associated SNPs were identified by mrMLM with the critical log of odds (LOD) score of 3.0 (*p* = 0.0002) (Wang et al., 2016). The QTNs were named using the nomenclature described by McCouch et al. (1997). For example, *q-C16:0-A03-1* indicated the first locus located on chromosome A03 associated with palmitic acid.

### Candidate Gene Prediction

Candidate genes were identified using significant SNP markers, which were detected using mrMLM (Wang et al., 2016). The association regions and 100-kb region upstream or downstream of peak SNPs associated with fatty acid content were identified based on the physical distance of chromosomes of significant associated-trait SNPs in the *B. napus* “Darmor-*bzh*” genome (version 4.1; Chalhoub et al., 2014). Subsequently, putative candidate genes were predicted according to the annotation of the SNP-tagged genome regions and confirmed by BlastP searches against the *Arabidopsis* genome with an *E*-value ≤ 1E-10. The function of these candidate genes was further annotated using the Kyoto Encyclopedia of Genes and Genomes (KEGG) database (https://www.genome.jp/kegg/pathway.html). Highly-orthologous genes involved in fatty acid biosynthesis were analyzed further, which were defined as the environment-insensitive and -sensitive genes according to the frequency detected between the different ecological genotypes and environments. To identify the directed functional genes for fatty acid, sequences of these candidate genes, isolated from plants with higher- and lower fatty acid levels, were aligned using ClustalW (Thompson et al., 1994) implemented in Geneious 4.8.5 software (Biomatters, Auckland, New Zealand).

### Analysis of the Expression Profiles of Candidate Fatty Acid-Associated Genes

Total RNA was extracted from the seeds of *B. napus* cultivar Zhongshuang No. 11 (ZS11) at 15 developmental stages (3–49 days after pollination) using the RNAprep Pure Plant Kit (Tiangen Biotech, Beijing, China), following the manufacturer's instructions. The cDNA library construction and RNA sequencing were performed using Novogene Bioinformatics Technology (Beijing, China). Transcriptome sequencing datasets were deposited in the BioProject database (BioProject ID PRJNA358784). The data were analyzed as previously described (Qu et al., 2015), and the expression profiles of the candidate genes were quantified in terms of fragments per kilobase of exon per million mapped fragments (FPKM), using Cufflinks with default parameters (Trapnell et al., 2012). These transcriptome datasets were previously deemed suitable for selecting candidate genes (Zhou et al., 2017). Candidate genes were validated using RT-qPCR analysis. Three biological replicates and three technical replicates were performed on a CFX96 Real-Time PCR system (Bio-Rad, Laboratories, Hercules, CA, USA), and the expression levels of candidate genes were calculated using the 2^−ΔΔ*Ct*^ method (Zhou et al., 2017). Hence, the expression values of the 106 candidate genes were normalized by Log_2_ (expression values). Heatmaps of the candidate genes were drafted using HemI 1.0 (http://hemi.biocuckoo.org/). The specific primer sequences used in this study were obtained from the qPCR Primer Database (Lu et al., 2017) and are listed in Supplementary Table S3.

## Results

### Phenotypic Variation and Correlation Among Different Rapeseed Genotypes

**Figure 1:**
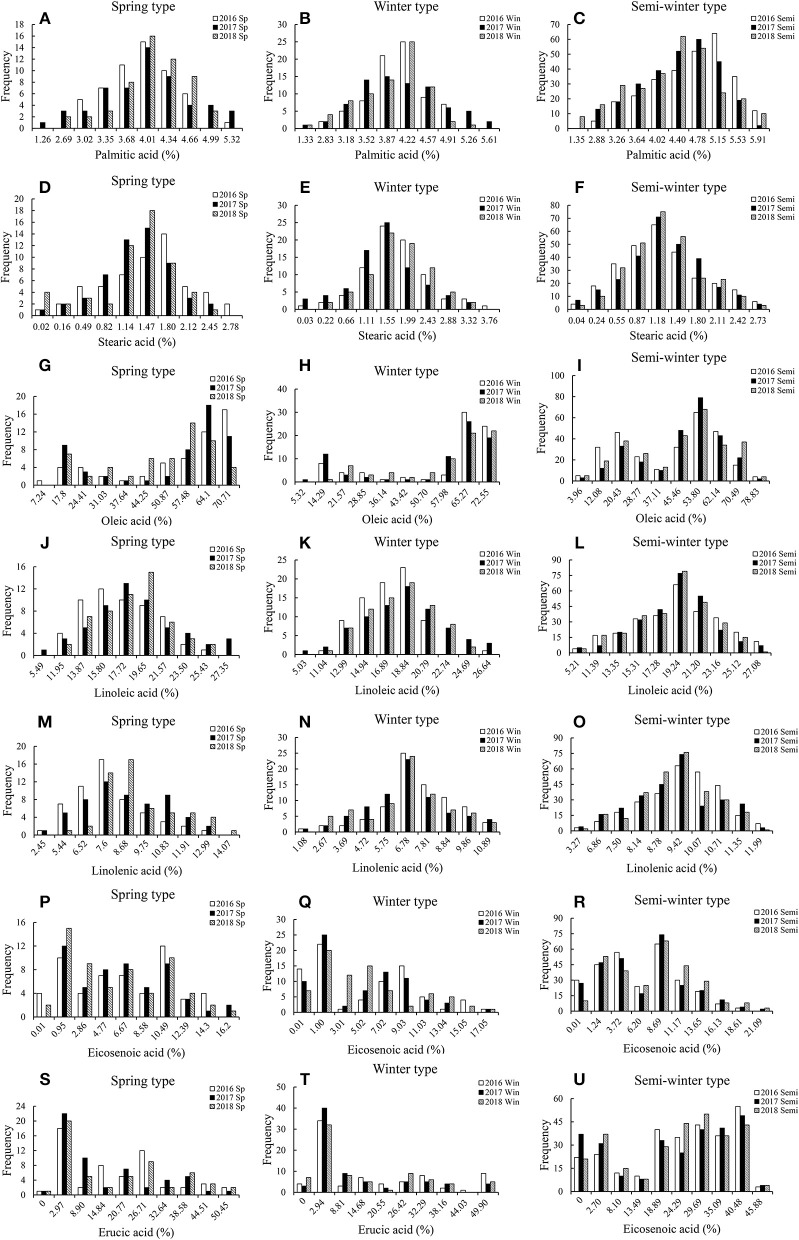
The frequency distribution of fatty acid contents in the different rapeseed accessions grown in different environments. The percentage indicates the proportion of the total dry weight of the seed represented by fatty acid composition. Sp, Spring-type rapeseed; Win, Winter-type rapeseed; Semi, Semi-winter-type rapeseed; 16, 17, and 18 represent the 2016, 2017, and 2018 growing seasons in Chongqing, China. **(A–C)** The frequency distribution of Palmitic acid contents in Spring-type, Winter-type, and Semi-winter-type rapeseed; **(D–F)** The frequency distribution of Stearic acid contents in Spring-type, Winter-type, and Semi-winter-type rapeseed; **(G–I)** The frequency distribution of Oleic acid contents in Spring-type, Winter-type, and Semi-winter-type rapeseed; **(J–L)** The frequency distribution of Linoleic acid contents in Spring-type, Winter-type, and Semi-winter-type rapeseed; **(M–O)** The frequency distribution of Linolenic acid contents in Spring-type, Winter-type, and Semi-winter-type rapeseed; **(P–R)** The frequency distribution of Eicosenoic acid contents in Spring-type, Winter-type, and Semi-winter-type rapeseed; **(S–U)** The frequency distribution of Erucic acid contents in Spring-type, Winter-type, and Semi-winter-type rapeseed.

Extensive variation in fatty acid content was observed between rapeseed plants of different genotypes grown in over 3 years (Table 1). The content of palmitic acid, stearic acid and linolenic acid varied slightly among the different ecotypic rapeseed accession at different years. For example, the mean palmitic acid content varied from 2.53 to 5.49% in spring rapeseed, 2.66 to 5.78% in winter rapeseed, and 2.69 to 6.10% in semi-winter rapeseed. The stearic acid content varied from 0.24 to 2.94% in spring rapeseed, 0.05 to 3.98% in winter rapeseed, and 0.09 to 2.89% in semi-winter rapeseed. The linolenic acid content varied from 4.90 to 14.61% in spring rapeseed, 2.15 to 11.41% in winter rapeseed, and 6.54 to 12.31% in semi-winter rapeseed. However, considerable quantitative variation was found for the content of oleic acid, linoleic acid, eicosenoic acid, and erucic acid. For instance, the mean oleic acid content ranged from 14.49 to 72.21% in spring rapeseed, 10.65 to 76.19% in winter rapeseed, and 7.91 to 83.00% in semi-winter rapeseed; the linoleic acid content ranged from 10.98 to 28.32%, 10.07 to 27.61%, and 10.41 to 28.07% in spring, winter, and semi-winter rapeseed, respectively, the eicosenoic acid content were ranged from 1.92 to 17.16%, 1.12 to 18.05%, and 0.22 to 22.34% in spring, winter and semi-winter rapeseed, respectively, and the erucic acid content ranged from 0 to 53.41 μmol g^−1^, 0 to 52.83 μmol g^−1^, and 0 to 48.58 μmol g^−1^ in spring, winter and semi-winter rapeseed, respectively (Table 1). Moreover, the largest CV (coefficient of variation) was found among the oleic acid, eicosenoic acid, and erucic acid content in different ecotypic rapeseed at different environments, ranging from 25.33 to 40.62, 50.00 to 183.00%, and 54.28 to 199.83%, respectively (Table 1), indicating that extensive variation was widely detected in the panel of accessions. In addition, the phenotypic values of fatty acid content were displayed among the ecotypic rapeseed accessions at different years (Figures 1A–U, Table 1). Of these, the palmitic acid, stearic acid, linoleic acid, and linolenic acid content were normally distributed, but the eicosenoic, oleic, and erucic acids content were skewed for three genotypic populations in different years (Figures 1A–U). Plants with higher oleic acid content were more common than those with lower content for each ecological type of rapeseed (Figures 1G–I). Analysis of variance (ANOVA) was performed among the spring, winter, and semi-winter rapeseed ecological types in different years, and showed that genotype and environment have significant effects on the fatty acid content of rapeseed (Table 1).

**Table 1:** Statistical analysis of fatty acid content in different ecological types of rapeseed grown in different environments.

**Fatty acid**	**Env**.	**Min**.	**Max**.	**Mean ± SD**	**CV(%)**	**Skewness**	**Kurtosis**	***F_***G***_***	***F_***E***_***
Palmitic acid	16Sp	2.93	5.44	3.92 ± 0.07	13.27	0.15	0.29	7.65^**^	4.82^*^
	17Sp	2.53	5.49	3.99 ± 0.09	17.29	0.05	−0.19		
	18Sp	3.03	5.14	4.18 ± 0.07	10.77	−0.36	0.19		
	16Win	2.77	5.06	4.04 ± 0.06	12.13	−0.35	0.18	6.81^**^	3.38^*^
	17Win	2.66	5.78	4.09 ± 0.08	16.14	0.20	−0.45		
	18Win	2.92	5.15	4.12 ± 0.05	10.44	−0.26	0.26		
	16Semi	3.19	6.10	4.89 ± 0.07	12.07	−0.80	0.64	7.10^**^	26.45^**^
	17Semi	2.86	5.29	4.38 ± 0.06	14.16	−0.75	−0.46		
	18Semi	2.69	5.04	4.20 ± 0.06	14.52	−0.93	−0.30		
Stearic acid	16Sp	0.92	2.94	1.85 ± 0.06	13.78	0.13	−0.07	5.57^**^	39.73^**^
	17Sp	1.04	1.96	1.42 ± 0.03	15.49	0.43	−0.21		
	18Sp	0.24	0.79	0.33 ± 0.03	13.64	−0.01	−0.69		
	16Win	0.70	3.98	1.86 ± 0.07	13.87	0.78	1.04	9.62^**^	17.99^**^
	17Win	0.55	3.46	1.47 ± 0.05	12.65	1.16	3.42		
	18Win	0.05	0.80	0.36 ± 0.03	58.33	0.00	−0.90		
	16Semi	0.09	1.06	0.62 ± 0.03	13.87	−0.68	0.23	8.97^**^	26.96^**^
	17Semi	1.05	2.89	2.08 ± 0.05	12.60	−0.45	−0.81		
	18Semi	0.58	2.83	1.91 ± 0.05	16.18	−0.68	−0.25		
Oleic acid	16Sp	14.49	74.02	56.37 ± 2.53	33.30	−1.22	−0.10	9.39^**^	309.73^**^
	17Sp	14.69	72.21	53.16 ± 2.65	36.98	−1.15	−0.36		
	18Sp	23.09	69.20	50.29 ± 1.86	25.33	−0.80	−0.40		
	16Win	10.97	76.03	56.49 ± 2.34	36.38	−1.27	−0.04	7.33^**^	179.39^**^
	17Win	10.65	76.19	53.73 ± 2.45	39.96	−1.08	−0.53		
	18Win	11.78	76.17	52.24 ± 1.99	30.88	−1.07	−0.20		
	16Semi	10.66	71.37	52.89 ± 1.73	27.15	−1.75	1.75	9.97^**^	269.64^**^
	17Semi	14.12	73.65	52.94 ± 1.88	34.91	−0.95	−0.77		
	18Semi	7.91	83.00	49.29 ± 2.11	40.62	−0.87	−0.77		
Linoleic acid	16Sp	11.68	26.15	17.38 ± 0.42	17.89	0.50	−0.09	8.67^**^	3.57^*^
	17Sp	10.98	28.32	18.76 ± 0.54	21.54	0.37	−0.20		
	18Sp	11.20	24.41	18.13 ± 0.45	17.21	−0.27	−0.52		
	16Win	11.34	27.61	17.13 ± 0.31	15.70	0.52	1.75	5.30^**^	3.21^*^
	17Win	10.07	26.87	17.87 ± 0.44	21.66	0.24	−0.54		
	18Win	10.92	23.10	17.31 ± 0.33	15.60	−0.05	−0.64		
	16Semi	12.66	28.07	22.34 ± 0.44	16.47	−0.72	0.10	5.57^**^	69.05
	17Semi	11.34	22.86	17.92 ± 0.29	15.85	−0.73	−0.51		
	18Semi	10.41	21.42	16.79 ± 0.29	16.62	−0.72	−0.64		
Linolenic acid	16Sp	4.90	10.04	7.39 ± 0.16	16.37	0.09	−0.55	6.82^**^	35.98^**^
	17Sp	6.73	12.15	9.12 ± 0.16	12.83	0.23	−0.09		
	18Sp	5.95	14.61	9.34 ± 0.22	15.95	0.51	2.45		
	16Win	2.15	11.41	7.45 ± 0.19	12.82	−0.15	0.62	7.16^**^	23.34^**^
	17Win	6.26	11.36	8.75 ± 0.12	12.11	−0.08	−0.35		
	18Win	5.60	11.13	8.79 ± 0.15	13.42	−0.03	0.02		
	16Semi	8.31	12.31	10.02 ± 0.11	9.08	0.36	−0.04	6.85^**^	35.01^**^
	17Semi	6.54	12.06	8.84 ± 0.11	12.78	0.30	−0.25		
	18Semi	6.88	11.69	8.84 ± 0.10	10.52	0.70	0.92		
Eicosenoic acid	16Sp	1.92	17.16	3.58 ± 0.72	50.00	1.20	0.09	9.27^**^	9.41^**^
	17Sp	2.31	17.03	3.10 ± 0.68	63.55	1.35	0.48		
	18Sp	2.91	16.30	6.96 ± 0.54	53.30	0.92	−0.25		
	16Win	1.12	18.05	3.07 ± 0.58	166.12	1.35	0.39	4.76^**^	12.92^**^
	17Win	2.01	16.81	2.47 ± 0.51	183.00	1.54	0.98		
	18Win	2.21	16.95	6.09 ± 0.45	60.10	1.17	0.32		
	16Semi	1.01	15.54	4.51 ± 0.43	79.60	1.56	1.70	7.33^**^	6.77^**^
	17Semi	0.78	19.44	4.88 ± 0.61	122.75	0.96	−0.52		
	18Semi	0.22	22.34	7.42 ± 0.68	86.52	0.69	−0.80		
Erucic acid	16Sp	2.42	53.41	25.82 ± 4.00	69.25	0.06	−1.50	7.99^**^	9.618^**^
	17Sp	1.19	52.77	13.42 ± 1.95	101.64	1.22	0.34		
	18Sp	0.00	36.13	10.38 ± 1.53	100.96	1.26	0.28		
	16Win	3.04	52.83	32.92 ± 3.73	54.28	−0.41	−1.41	9.35^**^	28.95^**^
	17Win	0.24	40.70	10.90 ± 1.40	107.34	1.30	0.31		
	18Win	0.00	38.61	10.15 ± 1.54	123.05	1.11	−0.37		
	16Semi	0.00	37.65	5.82 ± 1.40	199.83	1.90	1.93	6.24^**^	35.21^**^
	17Semi	0.00	42.92	10.11 ± 1.57	152.92	1.11	−0.62		
	18Semi	0.00	48.58	12.15 ± 1.77	138.52	1.01	−0.74		

*Env., environment; Min., Minimum; Max., Maximum; SD, standard deviation; CV, coefficient of variation; Sp, Spring-type rapeseed; Win, Winter-type rapeseed; Semi, Semi-winter-type rapeseed; 16, 17, and 18 represent the 2016, 2017, and 2018 growing seasons in Chongqing, China, respectively. F_G_ and F_E_: the F-values for genotypes and environments, respectively*.

* and

** *: the 0.05 and 0.01 levels of significance, respectively*.

### Genome-Wide Association Analysis of Fatty Acid via mrMLM

For palmitic acid (C16:0) content, 11 QTNs were detected on chromosomes A01, A03, A04, A06, A07, A08, C01, and C03, respectively (Table 2). Of these, three consensus QTNs (*q-C16:0-A06, q-C16:0-A08-2*, and *q-C16:0-C03-2*) were commonly detected for palmitic acid among different ecotypic rapeseed and ecotypic rapeseed cultivated in different years, providing useful information for searching for candidate genes associated with palmitic acid biosynthesis. However, *q-C16:0-A01, q-C16:0-A04, q-C16:0-A06*, and *q-C16:0-A07* were mainly found in the spring-type and all 3 years, and other QTNs were detected among different ecotypic rapeseed and years (Table 2).

**Table 2:** Quantitative trait nucleotides (QTNs) associated with fatty acid content in *B. napus* accessions grown in different environments.

**QTN**	**Chr**	**SNP associated**	**Position (bp)**	**–log_**10**_(P)**	**Environment**
***q-C16:0-A01***	A01	Bn-A01-p22085117	18711173	8.54	16Sp, 17Sp, 18Sp
***q-C16:0-A03-1***	A03	Bn-A03-p20145024	19008703	9.93	17Sp, 17Semi
***q-C16:0-A03-2***	A03	Bn-A03-p28560659	27022904	5.13	16Win, 17Sp
*q-C16:0-A04*	A04	Bn-A04-p14687930	15158346	6.72	16Sp, 17Sp, 18Sp
*q-C16:0-A06*	A06	Bn-A06-p16071214	17559622	5.68	16Sp, 17Sp, 18Sp
*q-C16:0-A07*	A07	Bn-A07-p10430301	11624458	7.78	17Sp, 18Sp
*q-C16:0-A08-1*	A08	Bn-scaff_16110_1-p214256	5357890	7.78	16Win, 17Sp, 18Sp, 18Semi
*q-C16:0-A08-2*	*A08*	Bn-A08-p13379983	11124385	7.70	16Win, 16Semi, 17Sp, 17Semi, 18Semi, 18Win
*q-C16:0-C01*	C01	Bn-A01-p12593802	16875948	15.26	17Win, 18Sp
*q-C16:0-C03-1*	C03	Bn-scaff_17298_1-p1471882	23560777	6.12	16Win, 17Win
*q-C16:0-C03-2*	C03	Bn-scaff_15794_3-p108033	55728615	8.09	16Sp,16Win, 16Semi, 17Sp, 17Semi, 18Semi
***q-C18:0-A03-1***	A03	Bn-A03-p21942870	20741914	6.65	16Semi, 18Win
***q-C18:0-A03-2***	A03	Bn-A03-p27339890	25582011	10.02	16Win, 17Sp
*q-C18:0-A06*	A06	Bn-A06-p6341389	5792362	5.22	17Sp, 18Sp
*q-C18:0-A08*	A08	Bn-A08-p10068904	8070062	5.39	16Win, 16Semi, 17Sp, 17Semi, 18Semi, 18Win
*q-C18:0-A09-1*	A09	Bn-A09-p3234323	3135040	5.30	16Semi, 17Sp, 18Win
*q-C18:0-A09-2*	A09	Bn-A09-p7329993	5542359	7.85	16Sp, 17Sp, 17Win
***q-C18:0-A10***	A10	Bn-A10-p13965313	13956813	11.41	16Sp, 17Sp
*q-C18:0-C03-1*	C03	Bn-scaff_17298_1-p779577	23106957	5.67	16Win, 17Sp, 17Win
*q-C18:0-C03-2*	C03	Bn-scaff_17457_1-p493971	53921047	6.00	17Semi, 18Sp
*q-C18:1-A01-1*	A01	Bn-A01-p2825565	2327566	8.27	16Semi, 18Sp
***q-C18:1-A01-2***	A01	Bn-A01-p26369651	20893064	11.54	17Sp, 18Sp
*q-C18:1-A02-1*	A02	Bn-A02-p10591779	7458917	7.37	16Semi, 18Sp, 18Win
***q-C18:1-A02-2***	A02	Bn-A02-p21061002	18906336	11.48	16Sp, 18Sp
***q-C18:1-A02-3***	A02	Bn-A02-p22386317	20775741	5.74	17Sp, 18Sp
***q-C18:1-A03***	A03	Bn-A03-p20369417	19241578	6.16	17Semi, 18Semi
***q-C18:1-A05***	A05	Bn-A05-p20425452	18636249	11.54	17Sp, 18Sp
*q-C18:1-A08-1*	A08	Bn-A08-p4077507	3476858	7.15	16Semi, 17Semi, 18Win
*q-C18:1-A08-2*	A08	Bn-A08-p7814432	6786988	7.38	16Semi, 17Semi, 18Semi, 18Win
*q-C18:1-A08-3*	A08	Bn-A08-p10068904	8070062	12.15	16Sp, 16Win, 16Semi, 17Sp, 17Win, 17Semi, 18Sp, 18Win, 18Semi
*q-C18:1-A08-4*	A08	Bn-A08-p12820786	10587675	13.42	16sp, 16Win, 16Semi, 17sp, 17Win, 17Semi, 18Sp, 18Semi
*q-C18:1-A08-5*	A08	Bn-scaff_24726_1-p33555	14029706	8.52	16Sp, 17Sp
*q-C18:1-A09*	A09	Bn-A09-p3051349	2971334	7.53	16Sp, 16Win, 17Semi, 18Win, 18Semi
*q-C18:1-C01*	C01	Bn-scaff_21015_1-p34786	32559311	13.22	16Sp, 16Semi, 18Sp
***q-C18:1-C02***	C02	Bn-scaff_16139_1-p1277806	45267495	8.71	16Sp, 16Win, 17Sp, 17Win, 18Semi
*q-C18:1-C03*	C03	Bn-scaff_15794_3-p89999	55717350	10.88	16Win, 16Sp, 16Semi, 17Sp, 17Win, 18Win
*q-C18:1-C04*	C04	Bn-scaff_16394_1-p1090896	32408105	6.18	17Sp, 17Semi, 18Sp
*q-C18:1-C05*	C05	Bn-scaff_20901_1-p1505546	2515848	11.54	16Semi, 18Sp
*q-C18:1-C07*	C07	Bn-scaff_16069_1-p431757	36777489	8.45	16Win, 17Sp, 18Sp, 18Win
*q-C18:1-C08*	C08	Bn-scaff_16361_1-p2793822	30283886	12.95	16Semi, 17Sp, 17Win, 18Sp
*q-C18:1-C09*	C09	Bn-scaff_16456_1-p415818	35068732	9.32	17Win, 18Sp
*q-C18:2-A01*	A01	Bn-A01-p4167795	3847687	7.15	17Semi, 18Sp
***q-C18:2-A03***	A03	Bn-A03-p20369417	19241578	7.04	17Semi, 17Sp
*q-C18:2-A04*	A04	Bn-A04-p14687930	15158346	8.26	17Sp, 18Sp
***q-C18:2-A06***	A06	Bn-A06-p22331680	21365690	5.08	17Sp, 18Sp
*q-C18:2-A07*	A07	Bn-A07-p14682292	22343999	6.88	17Sp, 18Win
*q-C18:2-A08-1*	A08	Bn-scaff_16110_1-p214256	5357890	12.18	16Semi, 17Sp, 17Semi
*q-C18:2-A08-2*	A08	Bn-A08-p14351709	12051686	12.18	16Semi, 17Sp, 17Semi, 18Semi, 18Win
*q-C18:2-A09*	A09	Bn-A09-p36112515	33233968	7.01	16Sp, 18Sp
***q-C18:2-A10***	A10	Bn-A10-p14179334	14175178	15.95	16Sp, 17Sp
*q-C18:2-C01*	C01	Bn-A08-p9268915	32559113	7.22	16Win, 18Sp
*q-C18:2-C03*	C03	Bn-scaff_15794_3-p108033	55728615	7.06	16Semi, 18Win, 17Semi, 18Semi
*q-C18:2-C05*	C05	Bn-scaff_16414_1-p863783	1091070	6.60	17Sp, 18Sp
*q-C18:2-C07*	C07	Bn-scaff_15705_1-p2274493	35279701	5.25	18Sp, 18Win
*q-C18:2-C09*	C09	Bn-scaff_18944_1-p566719	19915878	12.18	16Sp, 17Sp
*q-C18:3-A01-1*	A01	Bn-A01-p5243181	4826424	13.86	17Semi, 18Semi
*q-C18:3-A01-2*	A01	Bn-A01-p15090383	12600997	10.52	18Semi, 18Sp
***q-C18:3-A01-3***	A01	Bn-A01-p24431478	20229832	13.03	16Sp, 18Semi
*q-C18:3-A02-1*	A02	Bn-scaff_15714_1-p1537912	929885	12.25	17Win, 18Semi
***q-C18:3-A02-2***	A02	Bn-A02-p18101171	17261238	15.65	18Sp, 18Semi
*q-C18:3-A03-1*	A03	Bn-A03-p7011746	6295737	10.52	18Semi, 18Sp
*q-C18:3-A03-2*	A03	Bn-A03-p16162908	15257414	13.69	17Semi, 18Sp, 18Semi
***q-C18:3-A03-3***	A03	Bn-A03-p23609377	22177215	8.3	18Semi, 18Sp
*q-C18:3-A04-1*	A04	Bn-A04-p2765547	2466391	15.18	18Sp, 18Semi
*q-C18:3-A04-2*	A04	Bn-A04-p7629926	8963652	8.82	18Sp, 18Semi
*q-C18:3-A04-3*	A04	Bn-A04-p15296217	15753636	11.33	18Semi, 18Sp
*q-C18:3-A05-1*	A05	Bn-A05-p461633	571525	6.67	17Semi, 18Semi
*q-C18:3-A05-2*	A05	Bn-A05-p10939740	9532568	12.67	18Sp, 18Semi
***q-C18:3-A05-3***	A05	Bn-A05-p14206169	16030064	14.04	18Sp, 18Semi
*q-C18:3-A06-1*	A06	Bn-A06-p73924	60018	15.95	18Semi, 18Sp
*q-C18:3-A06-2*	A06	Bn-A06-p5535537	5007675	6.51	18Sp, 18Semi
***q-C18:3-A06-3***	A06	Bn-A06-p22331680	21365690	13.54	17Semi, 18Sp, 18Semi, 18Win
*q-C18:3-A07-1*	A07	Bn-Scaffold012966-p76	12552424	14.04	18Sp, 18Semi
*q-C18:3-A07-2*	A07	Bn-scaff_19937_1-p20028	21340943	8.18	17Semi, 18Semi, 18Sp
*q-C18:3-A08-1*	A08	Bn-A08-p2274232	1778991	10.52	18Sp, 18Semi
*q-C18:3-A08-2*	A08	Bn-A08-p6828857	5776774	6.55	16Sp, 17Semi, 18Semi
*q-C18:3-A08-3*	A08	Bn-A08-p15239790	12798553	6.43	17Semi, 18Semi, 18Win
*q-C18:3-A08-4*	A08	Bn-A05-p8245454	17667610	8.73	18Win, 18Semi
*q-C18:3-A09-1*	A09	Bn-A09-p2323366	1519271	14.04	18Sp, 18Semi
***q-C18:3-A09-2***	A09	Bn-A09-p24113289	23069752	10.68	17Semi, 18Semi, 18Sp
*q-C18:3-A09-3*	A09	Bn-A09-p31492693	29184323	14.04	18Sp, 18Semi
*q-C18:3-A10-1*	A10	Bn-A10-p3909275	913569	13.5	16Sp, 17Win, 18Sp, 18Semi
*q-C18:3-A10-2*	A10	Bn-A10-p7118112	8703408	14.56	17Semi, 18Semi
***q-C18:3-A10-3***	A10	Bn-A10-p16837056	16640509	13.5	16Sp, 18Semi, 18Sp, 18Win
*q-C18:3-C01*	C01	Bn-scaff_15838_5-p850445	3684748	14.95	16Sp, 17Semi, 18Semi
*q-C18:3-C02*	C02	Bn-scaff_18675_1-p230717	22324250	15.18	17Win, 16Sp, 18Semi, 18Sp
*q-C18:3-C03*	C03	Bn-scaff_26505_1-p5590	28729268	14.88	17Win, 18Semi
*q-C18:3-C04-1*	C04	Bn-scaff_16564_1-p236601	11168988	9.57	17Semi, 18Semi
*q-C18:3-C04-2*	C04	Bn-scaff_15779_1-p94004	30153296	13.19	16Sp, 17Semi, 18Semi
*q-C18:3-C04-3*	C04	Bn-scaff_16139_1-p785412	43939995	12.25	17Semi, 18Semi, 18Sp
*q-C18:3-C05-1*	C05	Bn-scaff_20901_1-p1719394	2295884	5.68	17Semi, 18Semi
*q-C18:3-C05-2*	C05	Bn-Scaffold000324-p108	8698912	7.29	18Sp, 18Win
*q-C18:3-C05-3*	C05	Bn-scaff_16454_1-p884909	21537168	9.06	16Sp, 18Sp
*q-C18:3-C06-1*	C06	Bn-scaff_17454_1-p225095	8428072	7.31	18Semi, 18Sp
*q-C18:3-C06-2*	C06	Bn-scaff_23957_1-p175042	30652290	15.26	16Sp, 17Semi, 18Semi, 18Sp
*q-C18:3-C07-1*	C07	Bn-scaff_22310_1-p321188	7983992	8.36	17Semi, 18Sp, 18Semi
*q-C18:3-C07-2*	C07	Bn-scaff_19106_1-p463047	18601376	10.61	17Semi, 18Semi
*q-C18:3-C07-3*	C07	Bn-scaff_16110_1-p2168404	42696563	10.29	17Semi, 18Win, 18Semi
*q-C18:3-C08-1*	C08	Bn-scaff_16174_1-p1445094	23687956	6.74	17Semi, 18Semi
*q-C18:3-C08-2*	C08	Bn-scaff_16445_1-p2523413	34371202	15.11	16Sp, 17Win, 18Sp, 18Semi
*q-C18:3-C09-1*	C09	Bn-scaff_20903_1-p300819	16920322	8.82	18Sp, 18Semi
*q-C18:3-C09-2*	C09	Bn-scaff_16297_1-p392549	23679499	6.01	18Semi, 18Sp
*q-C18:3-C09-3*	C09	Bn-scaff_20972_1-p160691	32990956	13.3	18Sp, 18Semi
*q-C20:1-A01-1*	A01	Bn-A01-p4167795	3847687	15.18	16Semi, 18Sp
***q-C20:1-A01-2***	A01	Bn-A01-p26369651	20893064	14.54	17Sp, 18Sp
*q-C20:1-A02-1*	A02	Bn-A02-p11284285	8292886	10.66	16Sp, 18Sp, 18Win
***q-C20:1-A02-2***	A02	Bn-A02-p21061002	18906336	13.65	17Sp, 18Sp
*q-C20:1-A03-1*	A03	Bn-A03-p16565487	15689355	13.20	18Semi, 18Sp
***q-C20:1-A03-2***	A03	Bn-A03-p27337536	25579664	14.40	16Win, 17Win, 18Sp
*q-C20:1-A04*	A04	Bn-A04-p14687930	15158346	9.40	17Sp, 18Sp
*q-C20:1-A05-1*	A05	Bn-A05-p5100352	4920148	7.95	16Win, 18Sp
***q-C20:1-A05-2***	A05	Bn-A05-p20425452	18636249	14.54	17Sp, 18Sp
*q-C20:1-A06-1*	A06	Bn-A06-p853722	1082917	6.01	16Semi, 17Sp, 17Win, 18Sp
***q-C20:1-A06-2***	A06	Bn-A06-p21116438	20562931	6.04	17Sp, 18Sp
*q-C20:1-A07*	A07	Bn-A07-p20230189	21986980	5.54	16Semi, 18Sp
*q-C20:1-A08-1*	A08	Bn-A08-p2711497	2151791	8.08	16Semi, 18Sp, 18Semi, 18Win
*q-C20:1-A08-2*	A08	Bn-A08-p13066424	10878218	13.82	16Sp, 16Semi, 16Win,17Sp, 17Semi, 18Semi, 18Sp
***q-C20:1-A09-1***	A09	Bn-A09-p26874249	24934319	9.82	18Sp, 18Semi, 18Win
*q-C20:1-A09-2*	A09	Bn-A09-p35656352	32788000	5.92	17Sp, 17Win
***q-C20:1-A10-1***	A10	Bn-A10-p13965313	13956813	14.54	16Sp, 17Sp, 18Sp
*q-C20:1-C01*	C01	Bn-scaff_17827_1-p963588	7866768	9.07	17Sp, 18Sp
***q-C20:1-C02***	C02	Bn-scaff_16139_1-p1267317	45277206	5.60	16Semi, 17Win
*q-C20:1-C03-1*	C03	Bn-scaff_17636_1-p3673	38484538	6.81	16Win, 17Win
*q-C20:1-C03-2*	C03	Bn-scaff_15794_3-p89999	55717350	12.34	16Sp, 16Semi, 16Win, 17Sp, 17Win, 18Win
*q-C20:1-C04-1*	C04	Bn-scaff_16394_1-p987099	32288653	12.72	17Sp, 17Semi, 18Sp
*q-C20:1-C04-2*	C04	Bn-scaff_15585_1-p276555	44214027	14.54	17Sp, 18Sp
*q-C20:1-C05-1*	C05	Bn-scaff_21338_1-p467919	11924522	6.42	16Win, 18Sp
*q-C20:1-C05-2*	C05	Bn-scaff_22099_1-p251444	24830406	13.97	16Semi, 17Sp, 18Sp
*q-C20:1-C05-3*	C05	Bn-A07-p541617	36934827	14.54	16Win, 17Sp, 18Sp
*q-C20:1-C07*	C07	Bn-scaff_16069_1-p431757	36777489	6.14	16Semi, 18Sp, 18Win
*q-C20:1-C08*	C08	Bn-scaff_21269_1-p121333	36981334	14.40	16Win, 17Sp, 18Sp
*q-C20:1-C09-1*	C09	Bn-scaff_17174_1-p62030	13393631	14.54	16Semi, 18Sp
*q-C20:1-C09-2*	C09	Bn-scaff_16456_1-p453404	35037965	15.18	16Win, 17Sp, 18Sp
*q-C22:1-A01*	A01	Bn-A01-p2825565	2327566	8.45	16Sp, 16Win, 16Semi, 18Sp
*q-C22:1-A02-1*	A02	Bn-scaff_16565_1-p1062007	6923746	6.36	18Sp, 18Win
***q-C22:1-A02-2***	A02	Bn-A02-p22386317	20775741	7.59	17Sp, 18Sp
*q-C22:1-A03-1*	A03	Bn-A03-p1923025	1541003	5.54	17Win, 18Win
***q-C22:1-A03-2***	A03	Bn-A03-p20417630	19283838	6.46	16Win, 17Semi, 18Sp
***q-C22:1-A06***	A06	Bn-A06-p21501350	20200573	5.11	16Sp, 18Sp
*q-C22:1-A08*	A08	Bn-A08-p13066424	10878218	12.38	16Sp,16Win, 16Semi, 17Sp, 17Win, 17Semi, 18Sp, 18Win, 18Semi
*q-C22:1-A09-1*	A09	Bn-A09-p3029767	2949844	9.43	17Sp, 17Win, 18Win, 18Semi, 18Sp
***q-C22:1-A09-2***	A09	Bn-A09-p27109839	25110047	5.45	17Sp, 17Win, 18Sp
*q-C22:1-A10*	A10	Bn-A10-p5819027	5451194	10.32	16Sp, 18Sp
***q-C22:1-C02***	C02	Bn-scaff_16139_1-p1051077	45458213	5.84	17Semi, 18Semi, 18Win
*q-C22:1-C03*	C03	Bn-scaff_17457_1-p493971	53921047	6.70	16Sp, 16Win, 16Semi, 17Sp, 17Win, 18Win
*q-C22:1-C05*	C05	Bn-scaff_18181_1-p1691104	6103006	8.95	16Sp, 18Sp
*q-C22:1-C07*	C07	Bn-scaff_15705_1-p1673044	34945104	6.66	17Win, 18Win
*q-C22:1-C08-1*	C08	Bn-A08-p6162660	14268534	5.89	17Win, 18Win
*q-C22:1-C08-2*	C08	Bn-scaff_16361_1-p2793822	30283886	8.57	17Sp, 18Sp

*Chr, Chromosome; Sp, Spring-type rapeseed; Win, Winter-type rapeseed; Semi, Semi-winter-type rapeseed; 16, 17, and 18 represent the 2016, 2017, and 2018 growing seasons in Chongqing, China, respectively. QTNs with underline were overlapped with those detected by Qu et al. (2017), and QTNs with bold font were identified in at least two ecotypic rapeseed and/or environments*.

For stearic acid (C18:0) content, a total of 9 QTNs were resolved and distributed on A03, A06, A08, A09, A10, and C03, respectively (Table 3). Among them, two QTNs, *q-C18:0-A08*, and *q-C18:0-C03-2*, were detected in different ecotypic rapeseed and environments, and others were detected in different ecotypic rapeseed grown in at least two different environments.

**Table 3:** Summary of candidate genes associated with fatty acid biosynthesis in significant association regions.

**Gene type**	**Chr**.	**Candidate gene**	**Annotation gene**	**Function description**	**Pathway ID and description**	**References**
Environmental insensitive	A06	BnaA06g25470D^b^	AT2G17930	Phosphatidylinositol 3- and 4-kinase family protein with FAT domain		
	A06	BnaA06g25480D^b^	AT2G17930	Phosphatidylinositol 3- and 4-kinase family protein with FAT domain		
	A06	BnaA06g30370D^b^	AT5G48230	acetoacetyl-CoA thiolase 2 (ACAT2)	K00626: Fatty acid metabolism
	A06	BnaA06g30420D^b^	AT5G48140	Pectin lyase-like superfamily protein		
	A06	BnaA06g30430D^b^	AT5G48100	TRANSPARENT TESTA 10 (TT10)	K05909: laccase
	A06	BnaA06g30780D^b^	AT3G29670	HXXXD-type acyl-transferase family protein		
	A06	BnaA06g30790D^b^	AT3G29635	HXXXD-type acyl-transferase family protein		
	A06	BnaA06g30800D^b^	AT3G29635	HXXXD-type acyl-transferase family protein		
	A06	BnaA06g31040D^b^	AT3G29152	Bifunctional inhibitor/lipid-transfer protein/seed storage 2S albumin superfamily protein		
	A08	BnaA08g08190D^b^	AT2G44730	Alcohol dehydrogenase transcription factor Myb/SANT-like family protein		
	A08	BnaA08g08280D^b^	AT4G17483	alpha/beta-Hydrolases superfamily protein	K01074: Fatty acid elongation
	A08	BnaA08g08850D^b^	AT4G18550	alpha/beta-Hydrolases superfamily protein		Kim et al., 2011
	A08	BnaA08g09510D^b^	AT4G20840	FAD-binding Berberine family protein		
	A08	BnaA08g11130D^b^	AT4G34520	3-ketoacyl-CoA synthase 18 (KCS18)	K15397: Fatty acid elongation	Wang et al., 2008; Wu et al., 2008; Li et al., 2014
	A08	BnaA08g11140D^b^	AT4G34510	3-ketoacyl-CoA synthase 17 (KCS17)	K15397: Fatty acid elongation	Tresch et al., 2012
	A08	BnaA08g11440D^b^	AT4G33790	ECERIFERUM 4 (CER4)	K13356: Cutin, suberine and wax biosynthesis	Rowland et al., 2006
	A08	BnaA08g11650D^b^	AT4G34030	3-methylcrotonyl-CoA carboxylase (MCCB)	K01969: 3-methylcrotonyl-CoA carboxylase beta subunit	Ding et al., 2012
	A08	BnaA08g11810D^b^	AT4G33355	Bifunctional inhibitor/lipid-transfer protein/seed storage 2S albumin superfamily protein		
	A09	BnaA09g02110D^a^	AT3G27660	oleosin 4 (OLEO4)		
	A09	BnaA09g05070D^b^	AT5G23970	HXXXD-type acyl-transferase family protein		
	A09	BnaA09g05410D^b^	AT5G23260	TRANSPARENT TESTA16 (TT16)		Deng et al., 2012
	A09	BnaA09g06170D^b^	AT2G25710	holocarboxylase synthase 1 (HCS1)		Tasseva et al., 2004
	A09	BnaA09g50060D^b^	AT1G06090	Fatty acid desaturase family protein		Smith et al., 2013
	A09	BnaA09g50070D^a^	AT1G06090	Fatty acid desaturase family protein		Smith et al., 2013
	A09	BnaA09g50080D^b^	AT1G06090	Fatty acid desaturase family protein		Smith et al., 2013
	C02	BnaC02g42220D^b^	AT5G23280	TCP family transcription factor		Aguilar-Martínez and Sinha, 2013
	C02	BnaC02g42240D^b^	AT5G23260	TRANSPARENT TESTA16 (TT16)		Deng et al., 2012
	C02	BnaC02g42690D^b^	AT5G63770	diacylglycerol kinase 2 (DGK2)	K00901: Glycerolipid metabolism
	C02	BnaC02g42700D^b^	AT5G63770	Diacylglycerol kinase 2 (DGK2)		
	C02	BnaC02g42910D^b^	AT5G64080	Bifunctional inhibitor/lipid-transfer protein/seed storage 2S albumin superfamily protein		
	C03	BnaC03g60080D^b^	AT4G38620	myb domain protein 4 (MYB4)	K09422: transcription factor MYB, plant
	C03	BnaC03g65980D^b^	AT4G34520	3-ketoacyl-CoA synthase 18 (KCS18)	K15397: Fatty acid elongation	Wang et al., 2008; Wu et al., 2008; Li et al., 2014
	C03	BnaC03g66040D^b^	AT4G34510	3-ketoacyl-CoA synthase 17 (KCS17)	K15397: Fatty acid elongation	Tresch et al., 2012
	C03	BnaC03g66380D^b^	AT4G33790	ECERIFERUM 4 (CER4)	K13356: Cutin, suberine and wax biosynthesis	Rowland et al., 2006
Environmental sensitive	A01	BnaA01g03850D^a^	AT4G33020	ZIP9		
	A01	BnaA01g08120D^b^	AT4G28780	GDSL-like Lipase/Acylhydrolase superfamily protein		
	A01	BnaA01g26680D^b^	AT3G18570	Oleosin family protein		
	A01	BnaA01g29500D^b^	AT3G14220	GDSL-like Lipase/Acylhydrolase superfamily protein		
	A01	BnaA01g30080D^b^	AT3G13062	Polyketide cyclase/dehydrase and lipid transport superfamily protein		
	A01	BnaA01g30110D^b^	AT3G13040	myb-like HTH transcriptional regulator family protein		
	A01	BnaA01g31150D^b^	AT3G11170	Fatty acid desaturase 7 (FAD7)		Maeda et al., 2008
	A02	BnaA02g13010D^a^	AT1G67260	TCP1		
	A02	BnaA02g13270D^a^	AT1G77590	Long chain acyl-CoA synthetase 9 (LACS9)	K01897: Fatty acid biosynthesis	Jessen et al., 2015
	A02	BnaA02g13310D^a^	AT1G67730	Beta-ketoacyl reductase 1 (KCR1)	K10251: Fatty acid elongation, Biosynthesis of unsaturated fatty acids
	A02	BnaA02g27960D^b^	AT4G11850	Phospholipase D gamma 1 (PLDGAMMA1)	K01115: Glycerophospholipid metabolism
	A02	BnaA02g28150D^b^	AT3G26650	glyceraldehyde 3-phosphate dehydrogenase A subunit (GAPA)		
	A02	BnaA02g30320D^b^	AT5G13930	TRANSPARENT TESTA 4 (TT4)	K00660: Flavonoid biosynthesis
	A02	BnaA02g30340D^b^	AT5G13930	TRANSPARENT TESTA 4 (TT4)	K00660: Flavonoid biosynthesis
	A02	BnaA02g30560D^b^	AT5G49070	3-ketoacyl-CoA synthase 21 (KCS21)	K15397: Fatty acid elongation
	A03	BnaA03g02250D^a^	AT5G07870	HXXXD-type acyl-transferase family protein		
	A03	BnaA03g02290D^a^	AT5G07920	Diacylglycerol kinase1 (DGK1)	K00901: Glycerophospholipid metabolism
	A03	BnaA03g02360D^a^	AT5G60830	basic leucine-zipper 70 (bZIP70)		
	A03	BnaA03g02470D^a^	AT5G08330	TCP family transcription factor		
	A03	BnaA03g03980D^a^	AT5G12420	O-acyltransferase (WSD1-like) family protein		Kalscheuer and Steinbüchel, 2003
	A03	BnaA03g03990D^a^	AT5G12420	O-acyltransferase (WSD1-like) family protein		Kalscheuer and Steinbüchel, 2003
	A03	BnaA03g04000D^a^	AT5G12420	O-acyltransferase (WSD1-like) family protein		Kalscheuer and Steinbüchel, 2003
	A03	BnaA03g13590D^a^	AT2G29980	fatty acid desaturase 3 (FAD3)		Hu et al., 2006; Yang et al., 2012
	A03	BnaA03g31600D^a^	AT3G11170	fatty acid desaturase 7 (FAD7)		
	A03	BnaA03g39010D^b^	AT4G34520	3-ketoacyl-CoA synthase 18 (KCS18)		
	A03	BnaA03g39500D^b^	AT5G23260	TRANSPARENT TESTA16 (TT16)		
	A03	BnaA03g49040D^b^	AT4G28130	diacylglycerol kinase 6 (DGK6)	K00901:Glycerolipid metabolism
	A05	BnaA05g00690D^a^	AT2G47210	myb-like transcription factor family protein		
	A05	BnaA05g09070D^a^	AT2G34770	fatty acid hydroxylase 1 (FAH1)		Nagano et al., 2012
	A05	BnaA05g25210D^b^	AT5G40990	GDSL lipase 1 (GLIP1)		
	A05	BnaA05g25220D^b^	AT3G14225	GDSL-motif lipase 4 (GLIP4)		
	A07	BnaA07g14020D^a^	AT2G28630	3-ketoacyl-CoA synthase 12 (KCS12)		Kim et al., 2013
	A08	BnaA08g12780D^b^	AT4G30950	fatty acid desaturase 6 (FAD6)	K10255:Two-component system
	A08	BnaA08g12800D^b^	AT4G30950	fatty acid desaturase 6 (FAD6)		
	A08	BnaA08g14190D^b^	AT4G27030	fatty acid desaturase A (FADA)		
	A08	BnaA08g14200D^b^	AT4G27030	fatty acid desaturase A (FADA)		
	A09	BnaA09g10550D^a^	AT1G62640	3-ketoacyl-acyl carrier protein synthase III (KAS III)	K00648:Fatty acid biosynthesis	Katayoon et al., 2001
	A09	BnaA09g10680D^b^	AT1G62940	acyl-CoA synthetase 5 (ACOS5)		
	A10	BnaA10g19670D^b^	AT5G13930	TRANSPARENT TESTA 4 (TT4)		
	A10	BnaA10g24560D^b^	AT5G05960	Bifunctional inhibitor/lipid-transfer protein/seed storage 2S albumin superfamily protein		
	C01	BnaC01g12360D^a^	AT4G20870	fatty acid hydroxylase 2 (FAH2)		
	C01	BnaC01g22230D^a^	AT5G49555	FAD/NAD(P)-binding oxidoreductase family protein		
	C01	BnaC01g23310D^a^	AT3G51590	lipid transfer protein 12 (LTP12)		
	C04	BnaC04g27640D^b^	AT3G53100	GDSL-like Lipase/Acylhydrolase superfamily protein		
	C04	BnaC04g32530D^b^	AT5G40420	oleosin 2 (OLEO2)		
	C05	BnaC05g02350D^a^	AT1G04220	3-ketoacyl-CoA synthase 2 (KCS2)		
	C05	BnaC05g04810D^a^	AT2G30310	GDSL-like Lipase/Acylhydrolase family protein		
	C05	BnaC05g04820D^a^	AT2G24560	GDSL-like Lipase/Acylhydrolase family protein		
	C05	BnaC05g14920D^a^	AT1G74960	fatty acid biosynthesis 1 (FAB1)	K09458: Fatty acid biosynthesis
	C05	BnaC05g36500D^a^	AT3G16850	Pectin lyase-like superfamily protein		
	C06	BnaC06g08390D^a^	AT1G34790	transparent testa 1 (TT1)		Lian et al., 2017
	C07	BnaC07g05570D^a^	AT2G16280	3-ketoacyl-CoA synthase 9 (KCS9)	K15397: Fatty acid elongation	Kim et al., 2013
	C07	BnaC07g29950D^b^	AT5G24520	TRANSPARENT TESTA GLABRA 1 (TTG1)		
	C07	BnaC07g30210D^b^	AT5G24180	Lipase class 3-related protein		
	C07	BnaC07g41010D^a^	AT3G05970	long-chain acyl-CoA synthetase 6 (LACS6)	K01897:Fatty acid biosynthesis	Hsiao et al., 2014
	C07	BnaC07g41430D^a^	AT4G28570	Long-chain fatty alcohol dehydrogenase family protein		
	C07	BnaC07g42880D^a^	AT4G30720	FAD/NAD(P)-binding oxidoreductase family protein		
	C08	BnaC08g32310D^b^	AT3G62590	Alpha/beta-Hydrolases superfamily protein		
	C09	BnaC09g05650D^a^	AT5G62470	myb domain protein 96 (MYB96)		
	C09	BnaC09g20440D^a^	AT2G01180	Phosphatidic acid phosphatase 1 (PAP1)		
	C09	BnaC09g30320D^b^	AT5G54060	UDP-glucose:flavonoid 3-o-glucosyltransferase (UF3GT)	K17193: Anthocyanin biosynthesis

*Chr, Chromosome*.

a, b *The candidate genes for fatty acid content around the isolated and overlapped QTNs, respectively*.

For oleic acid (C18:1) content, 21 QTNs were detected and distributed throughout of the *B. napus* genome, including chromosomes A01, A02, A03, A05, A08, A09, C01, C02, C03, C04, C05, C07, C08, and C09, respectively (Table 2). Of these, seven QTNs (*q-C18:1-A08-3, q-C18:1-A08-4, q-C18:1-A09, q-C18:1-C03, q-C18:1-C04, q-C18:1-C08*, and *q-C18:1-C09*) were co-localized in the same genomic regions of A08, A09, C03, C04, C08, and C09 using mrMLM and the PCA+K model (Qu et al., 2017). These seven QTNs were considered the major candidate regions for oleic acid content.

For linoleic acid (C18:2) content, fourteen QTNs were detected and mapped on chromosomes A01, A03, A04, A06, A07, A08, A09, A10, C01, C03, C05, C07, and C09, respectively (Table 2). Of these, *q-C18:2-A08-2, q-C18:2-A09, q-C18:2-C03*, and *q-C18:2-C07* were identified in our previous research (Qu et al., 2017).

For linolenic acid (C18:3) content, a total 48 QTNs were found and covered almost the whole *B. napus* genome (Table 2). Among them, seven highly identical QTNs distributed on chromosome A01, A02, A05, A06, A08, A09, and C02, along with other minor loci could be identified in different ecotypic rapeseed accessions in at least 1 year of growth (Table 2).

For eicosenoic acid (C20:1) content, 30 QTNs were obtained, including seven QTNs that overlapped with previously published QTNs (Qu et al., 2017), and were distributed on chromosome A01, A04, A06, A08, C03, C07, and C09, respectively (Table 2). The novel loci for eicosenoic acid content also displayed marked variation among the different ecotypic rapeseeds and environments.

For erucic acid (C22:1) content, 16 QTNs were detected and mapped on chromosome A01, A02, A03, A06, A08, A09, A10, C02, C03, C05, C07, and C08, respectively (Table 2). Of these, two QTNs (*q-C22:1-A08* and *q-C22:1-C03*) had been widely considered as the major genetic regions of A08 and C03, consistent with the findings of published works (Li et al., 2014; Lee et al., 2015; Xu et al., 2015; Qu et al., 2017). In addition, two QTNs (*q-C22:1-A09-1* and *q-C22:1-C08-1*) associated with erucic acid content were also detected in different ecotypic rapeseed and environments (Table 2), indicating that mrMLM is a powerful and accurate tool to detect QTNs and estimate the effect of QTNs on complex traits.

**Figure 2:**
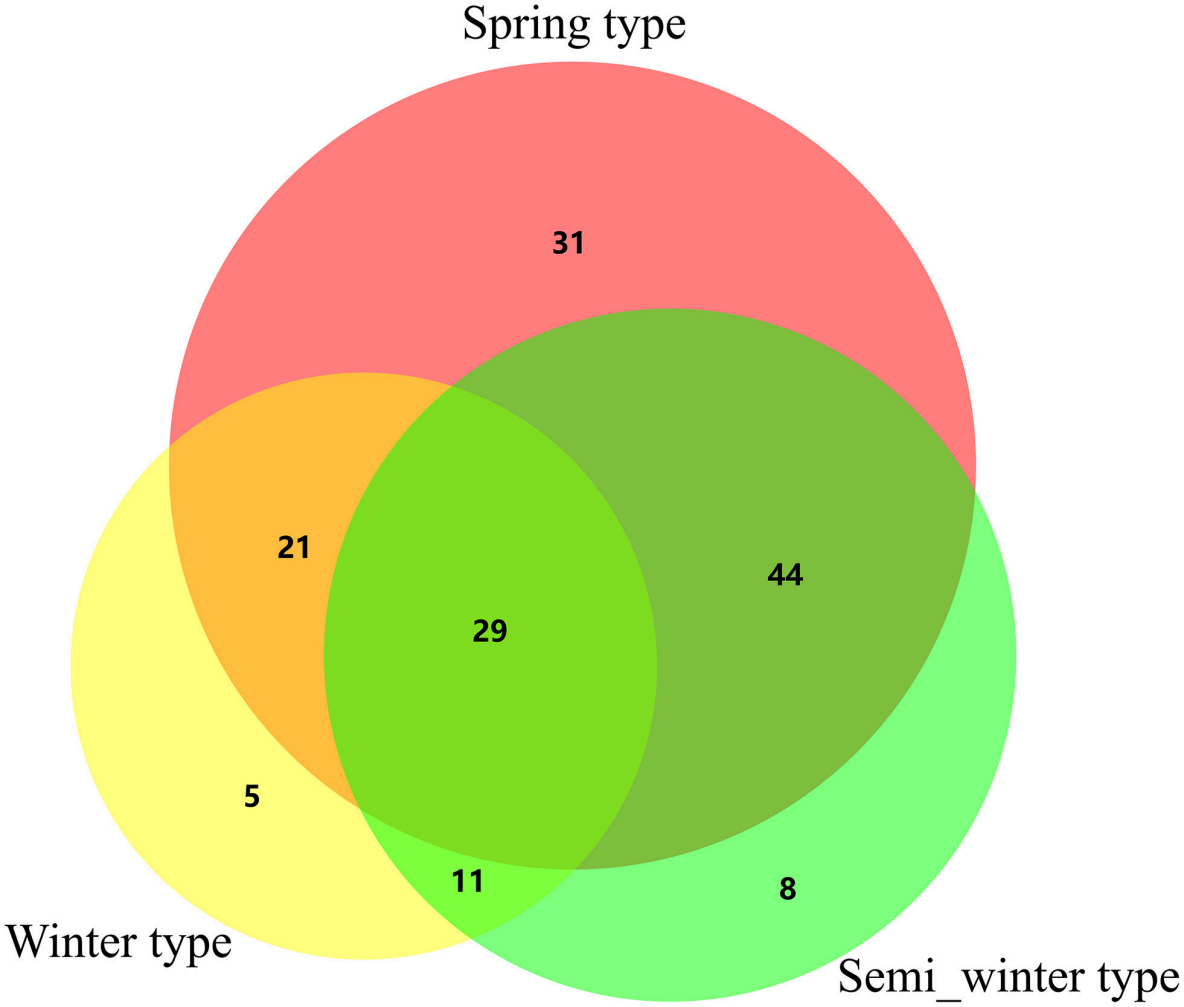
Venn diagram analysis of QTNs for fatty acids in different ecotypic rapeseed.

In all, 149 QTNs associated with fatty acid content were detected using mrMLM (Table 2, Supplementary Table S2), while only 62 associated regions were detected using the PCA + K model in TASSEL 5.2.1 (Qu et al., 2017). Among these, 34 QTNs were overlapped, including the association regions on A08 and C03, which had been widely reported previously (Wang et al., 2015; Liu et al., 2016; Qu et al., 2017), indicating that these results were credible and reproducible. In addition, 115 novel loci were identified for fatty acids via mrMLM compared with MLM (PCA+K; Table 2), indicating that a multi-locus random effect MLM method was better able to detect QTNs of complex quantitative traits. Furthermore, of these, 30.43% novel QTNs (35/115) were repeatedly detected for fatty acid content among different ecotypic rapeseed accessions and environments, located on chromosome A01, A02, A03, A05, A06, A09, A10, and C02, respectively (bold in Table 2), and other novel QTNs (80/115) were found for fatty acid content in a single environment. Among these QTNs, 29 were simultaneously detected in three ecotypic rapeseed, with greater QTN variation in spring and semi-winter rapeseed (Figure 2, Supplementary Table S2). Our results provideinsight into the mechanism underlying fatty acid composition, and lay the foundation for marker assisted selection in breeding projects for improved rapeseed genotypes with high oil quality and an ideal fatty acid composition.

### Identification of Candidate Genes

To predict candidate genes for loci significantly associated with fatty acid content, the reported and repeadly detected novel QTNs were used to confirm the genomic regions in the *B. napus* “Darmor v4.1” reference genome (Chalhoub et al., 2014). We identified five environment-insensitive and fifteen environment-sensitive association regions and screened for candidate genes within these regions. Subsequently, we extracted gene sequences within the GWAS peaks in candidate association regions, and identified 95 putative genes that possibly influence fatty acid content (Table 3). Of these, 63.16% candidate genes (60/95) were screened in the overlapping and repeatedly detected association regions, while the remaining genes (35/95) were detected on the single QTN regions (Table 3). Using peak SNPs on A08 and C03 (Bn-A08-p13066424 and Bn-scaff_15794_3-p89999, respectively), 9 and 4 candidate genes were selected in the association regions on each chromosome, respectively (Table 3). *BnaA08g11130D* and *BnaC03g65980D* are putative paralogs of 3-ketoacyl-CoA synthase 18 (*KCS18*), while *BnaA08g11140D* and *BnaC03g66040D* are putative paralogs of *KCS17* (Table 3), based on comparisons of the physical positions of genes associated with erucic acid traits in a GWAS (Wu et al., 2008; Li et al., 2014; Lee et al., 2015; Xu et al., 2015), indicating that there is a strong correlation between GWAS peak regions and candidate genes. In addition, the putative candidate genes were characterized and annotated, such as 3-methylcrotonyl-CoA carboxylase (*MCCB, BnaA08g11650D*), *TRANSPARENT TESTA*16 (*TT16, BnaA09g05410D*, and *BnaC02g42240D*), and *MYB4* (*BnaC03g60080D*), which might be environment-insensitive genes located on chromosome A06, A09, and C02 respectively (Table 3). Of these, 17 genes had been identified that might be involved in the fatty acid pathway, and 12 members were annotated for fatty acid metabolism in KEGG analysis (Table 3).

The expression of candidate genes in low-frequency association regions identified in this study were influenced by the genotype and environments, and 61 environment-sensitive genes were obtained by comparing these regions with the *B. napus* reference genome (Chalhoub et al., 2014), including *GDSL* (GDSL-like Lipase), *GAPA* (Glyceraldehyde 3-phosphate dehydrogenase A), *KCS21, FAD3, FAD7, FAD6* fatty acid biosynthesis 1 (*FAB1*), acyl-activating enzyme 17 (*AAE17*), long chain acyl-CoA synthetase 9 (*LACS9*), oleosin 2 (*OLEO2*), beta-ketoacyl reductase 1 (*KCR1*), and trigalactosyldiacylglycerol2 (*TGD2*) (Table 3). Of these, some genes (e.g., *TT16* and *TT1*) were predicted to be associated with oleic acid content in *B*. *napus* (Lian et al., 2017; Qu et al., 2017); however, novel loci were also identified, including *MYB67, OLEO2, KCS21, FAD3, KCR1, TT1*, and *TGD2* (Table 3). Among these genes, 12 putative gene members were identified in previous research, and 14 gene members were enriched in fatty acid pathways in the KEGG database (Table 3).

**Figure 3:**
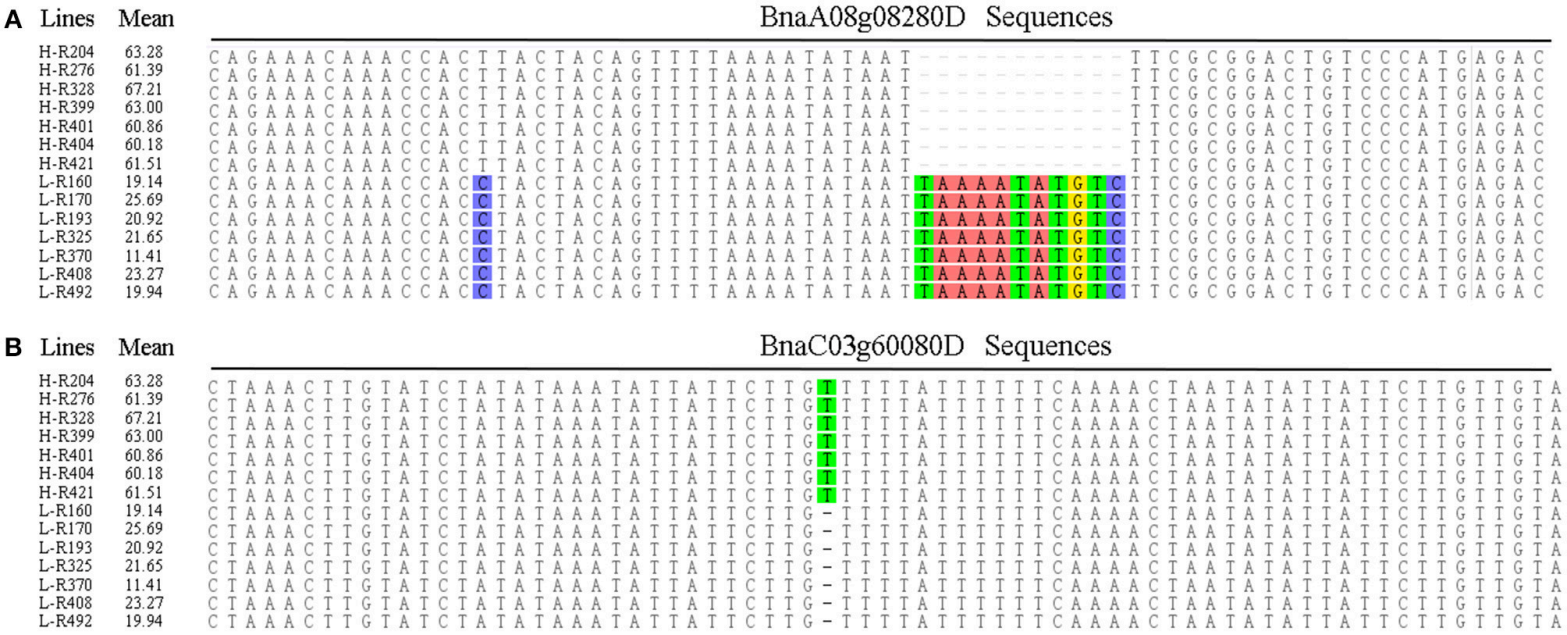
Multiple alignments of candidate gene sequences between the high- and low-oleic acid *B. napus* accessions. **(A)** BnaA08g08280D; **(B)** BnaC03g60080D. H-number and L-number indicates the high- and low-oleic acid *B. napus* accessions, respectively. The oleic aicd contents represents by the mean values.

Oleic acid is a monounsaturated fat beneficial for human health that contributes to the nutritional and economic value of rapeseed oil. To provide insight into the genetic control of oleic acid content, we therefore aligned 95 gene sequences from the different rapeseed accessions, and identified nucleotides in the intronic regions of *BnaA08g08280D* and *BnaC03g60080D* that show significant differences between the high- and low-oleic acid lines (Figure 3).

### Expression Patterns of Candidate Genes

**Figure 4:**
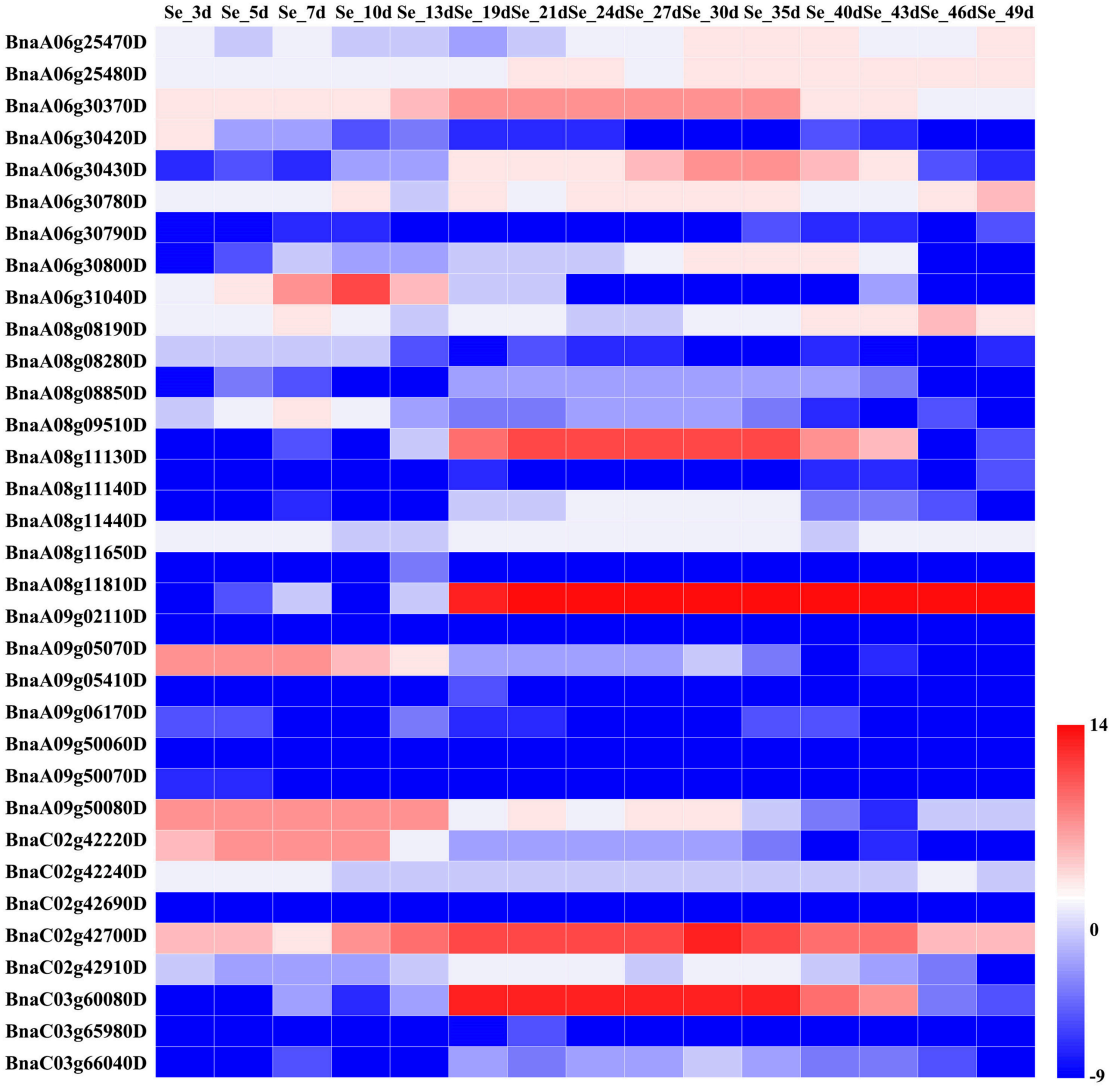
Comparative expression analysis of environment-insensitive genes associated with fatty acid content during seed development. The abbreviations above the heatmap indicate the different developmental stages of the seeds from *B*. *napus* ZS11 (defined in Supplementary Table S4). The expression values of the candidate genes were calculated using three biological replicates with three technical replicates and normalized by Log_2_ (mean expression values). The “scale” function in R was used to normalize the relative expression levels (*R* = log_2_/mean expression values). The heatmap was generated using Heatmap Illustrator 1.0 (HemI 1.0).

**Figure 5:**
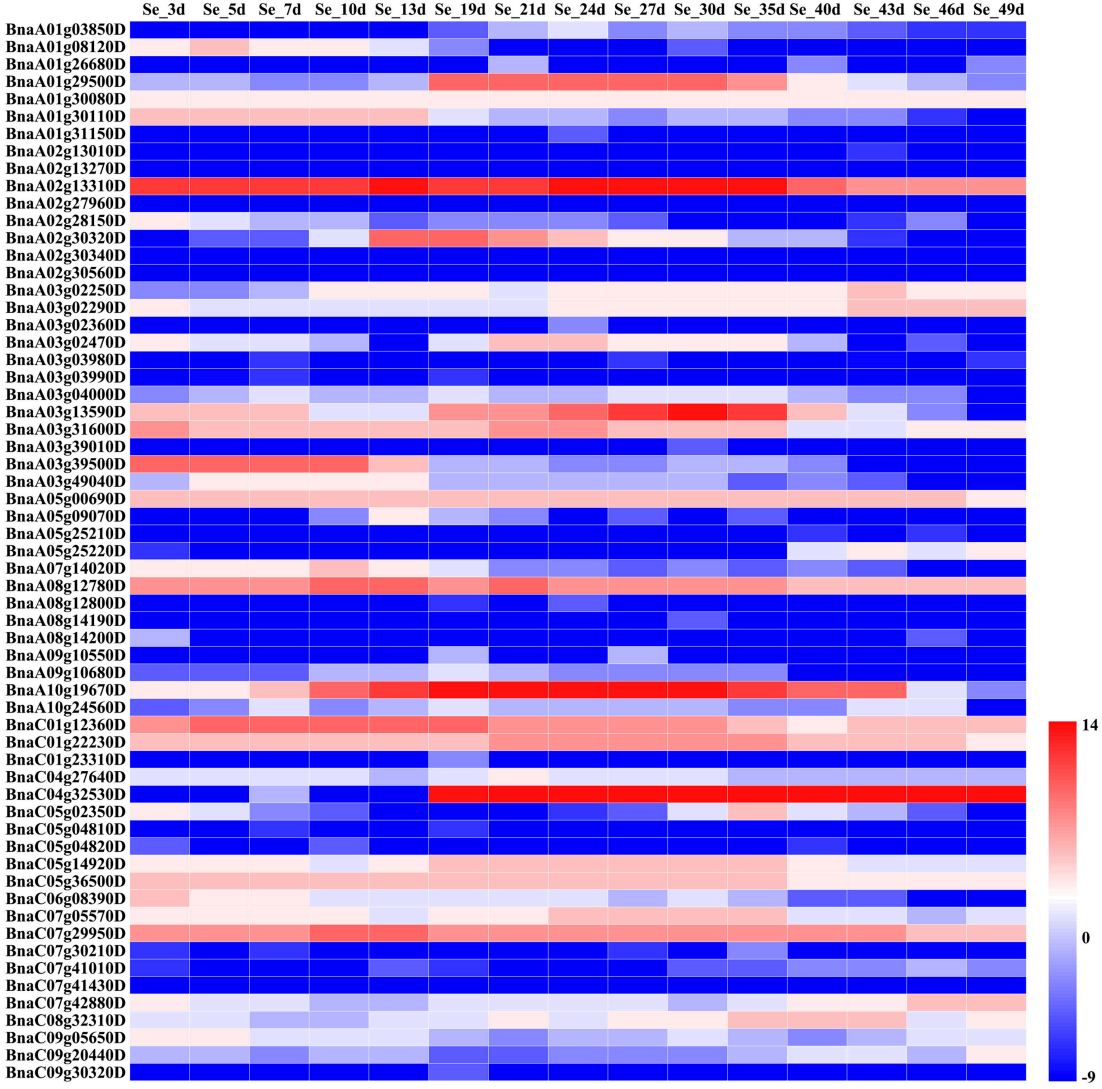
Comparative expression analysis of environment-sensitive genes associated with the fatty acid content during seed development. The abbreviations above the heatmap indicate the different developmental stages of the seeds from *B*. *napus* ZS11 (defined in Supplementary Table S5). The expression values of the candidate genes were calculated using three biological replicates with three technical replicates and normalized by Log_2_ (mean expression values). The “scale” function in R was used to normalize the relative expression levels (*R* = log_2_/mean expression values). The heatmap was generated using Heatmap Illustrator 1.0 (HemI 1.0).

We assessed the relative expression levels of the candidate genes during seed development of *B. napus* variety ZS11 (Figures 4, 5), which had a high oleic acid content and low erucic acid. The expression levels of the environment-insensitive genes showed no obvious variation during seed development, but *KCS18* (*BnaA08g11130D* and *BnaC03g65980D*), and *BnaC02g42910D* showed higher expression levels during the middle stages of seed development (Figure 4), indicating that they might contribute to the accumulation of oleic acid during the middle stages of seed development. In addition, *TT16* (*BnaA09g05410D* and *BnaC02g42240D*) and *BnaC02g42240D* were expressed at high levels in the early stages of seed development, while other genes showed low expression levels throughout seed development (Figure 4).

However, we found that the expression levels of the environment-sensitive genes varied throughout seed development (Figure 5). For example, *OLEO2* (*BnaC04g32530D*) was highly expressed in the middle and late stages of seed development, while the expression of *KCS9* (*BnaC07g05570D*) peaked in the early and middle stages (Figure 5). In addition, *BnaA01g29500D* and *TT4* (*BnaA02g30320D*) were mainly expressed in the middle stages of seed development, but *KCR1* (*BnaA02g13310D*) and *TTG1* (*BnaC07g29950D*) showed high expression levels throughout seed development (Figure 5). Furthermore, other genes displayed different patterns of expression throughout seed development.

## Discussion

In *B. napus*, seeds fatty acids are mainly composed of palmitic, stearic, oleic, linoleic, linolenic eicosenoic, and erucic acids, which determine the rapeseed oil quality. Enhancing the oleic acid content and quality of rapeseed through modifying its fatty acid composition has become an important breeding goal. However, previous studies identified the effect of putative fatty acid genes and the interaction of genotype and environment on the fatty acid content of rapeseed (Zhao, 2002; Zhao et al., 2005, 2008; Wen et al., 2015). Here, we report that fatty acid content also varies significantly among different rapeseed ecological types (spring, winter, and semi-winter rapeseed varieties) and environments (Figure 1, Table 1), indicating the complexity of the biosynthetic processes underlying fatty acid content in rapeseed. Interestingly, accessions with high oleic acid content were common amongst the different rapeseed ecological types (Figures 1G–I), possibly because these accessions are artificially selected in breeding projects aimed at producing rapeseed with high oleic acid content. Therefore, identifying the relationship between favorable alleles and environments will be beneficial for improving the fatty acid content of rapeseed.

With the development of genome sequencing and computational technologies, the Illumina Infinium *Brassica* 60K SNP array has been developed and widely used for the genome-wide association analysis of *B. napus* as well as the analysis of some trait-associated genomic regions and candidate genes (Li et al., 2014; Qian et al., 2014; Gajardo et al., 2015; Hatzig et al., 2015; Lee et al., 2015; Wei et al., 2015; Gacek et al., 2017; Qu et al., 2017). Furthermore, the MLM (Q+K and PCA+K) was found to be a powerful model for GWAS in these previous studies (Yu et al., 2006; Zhao et al., 2011; Xu et al., 2015; Li et al., 2016; Qu et al., 2017). In the present study, the mrMLM was employed for a GWAS, which is confirmed as a precise and powerful tool for analyzing phenotypic and genotypic information derived from numerous accessions and SNPs (Wang et al., 2016; Li et al., 2017). In the present study, 149 QTNs significantly associated with fatty acid content were identified using the mrMLM (Table 2, and Supplementary Table S3). Of these, 34 associated SNPs overlapped with those obtained using MLM (Qu et al., 2017), indicating that the association analysis was reliable; however, eight novel association regions containing 35 QTNs were simultaneously detected among different ecotypic rapeseed grown in different environments, strongly suggesting that mrMLM is more powerful to detect SNPs associated with complex traits than MLM in GWAS. In addition, 29 QTNs for fatty acids were simultaneously detected in spring, winter and semi-winter rapeseed (Figure 2), indicating that the orthologous genes for fatty acid might be better identified using these singinficant QTNs. Furthermore, more QTNs associated with fatty acids were identified from the sping and semi-winter type than in winter rapeseed (Figure 2), indicating that the fatty acids were associated with their genotype. These results might be helpful for elucidating the mechanism that determines fatty acid composition in *B. napus*.

In *B. napus*, fatty acid variation is controlled by multiple genes (Zhao et al., 2008; Wen et al., 2015). In this study, we categorized the candidate genes as either environment-insensitive or -sensitive genes, according to the published results and their detection frequency between the rapeseed genotypes grown in the different environments. A total of 95 candidate genes were identified with known functions in the fatty acid biosynthesis pathway. Of these, 34 were environment-insensitive genes (Table 3), including *KCS18*, which is known to play a crucial role in regulating erucic acid biosynthesis in *B. napus* (Wang et al., 2008; Wu et al., 2008; Li et al., 2014), and the putative *KCS18* paralogs *BnaA08g11130D* (Chromosome A08) and *BnaC03g65980D* (Chromosome C03), which are most highly expressed during the middle to late stages of seed development (Figure 4), suggesting that they are key genes regulating the accumulation of fatty acids in rapeseed oil. In addition, *CER4* encodes an alcohol-forming fatty acyl-coenzyme A reductase (Rowland et al., 2006), and *KCS17* is known to be involved in the biosynthesis of saturated fatty acids (Tresch et al., 2012) and would therefore be expected to be more highly expressed during seed development. We found that the putative orthologs of *KCS17* (*BnaA08g11140D* and *BnaC03g66040D*) and *CER4* (BnaC03g66380D) were associated with fatty acid content in *B. napus*, but the expression of *BnaA08g11140D* and *BnaC03g66040D*, putative *KCS17* paralogs, was downregulated during seed development (Figure 4), suggesting that functional segregation exists among the different paralogs of the ancestral *KCS17* gene, which has been reported previously in the *B. napus* genome (Chalhoub et al., 2014). In addition, the environment-insensitive genes, including *DGK2* (*BnaC02g42690D* and *BnaC02g42700D*), *HCS1* (*BnaA09g06170D*), *MYB4* (*BnaC03g60080D*), and *TT16* (*BnaA09g05410D* and *BnaC02g42240D*), might also been involved in fatty acid biosynthesis (Tasseva et al., 2004; Deng et al., 2012; Yang et al., 2012; Chen et al., 2013). Most of the environment-insensitive genes were steadily expressed throughout seed development (Figure 4), and these might be the major factors regulating oleic acid accumulation in *B. napus*. Furthermore, 12 environment-insensitive genes were enriched in the fatty acid biosynthesis pathway according to KEGG pathway analysis (Table 3). Importantly, the nucleotide sequences of *BnaA08g08280D* and *BnaC03g60080D* differed between the high- and low-oleic acid lines (Figure 3), but these differences were all located in the intronic regions. Furthermore, 61 environment-sensitive genes showed wide variation in expression during seed development (Table 3, Figure 5), and these could be divided into early, middle, and late expression genes, respectively. For example, *BnaA06g31040D* showed high expression levels in the early stages of seed development, *KCS9* (*BnaC07g05570D*) peaked at the early and middle stages, *OLEO2* (*BnaC04g32530D*) had high expression during the middle and late stages, and *BnaA01g29500D*, and *TT4* (*BnaA02g30320D*) expression markedly increased in the middle stages (Table 3, Figure 5). Several other candidate genes, including putative homologs of *FAD* (3, 6, and 7), *KCS* (9, 12, and 21), *KCR1*, and *LACS9*, were found to be associated with fatty acid biosynthesis (Peng et al., 2010; Tresch et al., 2012; Yang et al., 2012; Lai et al., 2017; Shi et al., 2017), but their contribution remains to be confirmed by further studies (Table 3). In addition, 14 gene members were involved in the fatty acid metabolism confirmed by KEGG database analysis (Table 3). However, there is no clear evidence indicating that these genes control the fatty acid content in *B*. *napus*.

In summary, 149 QTNs for fatty acid content (including 34 reported and 115 novel loci) were detected, strongly demonstrating that mrMLM is a powerful and suitable tool for detecting QTNs for fatty acid content in rapeseed. Among these putative candidate genes, 63.16% (60/95) and 36.84% (35/95) were distributed on overlapping and isolated QTNs, respectively. Based on the pervious reports, 29 genes are involved in the fatty acid biosynthesis, and 26 gene members were enriched in the fatty acid pathway by the KEGG pathway database, indicating that mrMLM is an accurate tool to estimate the effect of QTNs on complex traits. Whether these genes exert significant regulatory effects on the fatty acid content of the seeds remains to be investigated. Hence, further studies are needed. Our findings provide useful candidate genes for the marker-assisted selection and breeding of rapeseed lines with increased oleic acid content in the seed.

## Author Contributions

CMQ and JL conceived and designed the experiments; MG and XH conducted the experiments; ZX, XX, LJ, and KL collected and analyzed the data; SW, and LJ made sequence alignment; YL, LW, and RW carried out the field experiments; MG, MZ, and CLQ wrote the manuscript; JL and CMQ reviewed the manuscript.

### Conflict of Interest Statement

The authors declare that the research was conducted in the absence of any commercial or financial relationships that could be construed as a potential conflict of interest.

## Supplementary Material

Supplementary Figure S1 Subpopulations in the spring, winter, and semi-winter *B. napus* accessions, determined using a principal coordinate analysis (PCA).

Supplementary Table S1 List of rapeseed accessions used.

Supplementary Table S2 Sum of SNPs in candidate regions for fatty acid content in *B. napus* accessions grown in different environments.

Supplementary Table S3 Primers used to amplify candidate genes and reference genes via RT-qPCR analysis.

Supplementary Table S4 Heatmap of the expression levels of the *B. napus* environment-insensitive genes at different stages of seed development.

Supplementary Table S5 Heatmap of the expression levels of the *B. napus* environment-sensitive genes at different stages of seed development.
