# Supplementary figures and images for: Association Mapping Analysis of Fatty Acid Content in Different Ecotypic Rapeseed Using mrMLM
Source: Front Plant Sci. 2019 Jan 4;9:1872. doi: 10.3389/fpls.2018.01872 (PMC6328494; doi:10.3389/fpls.2018.01872)

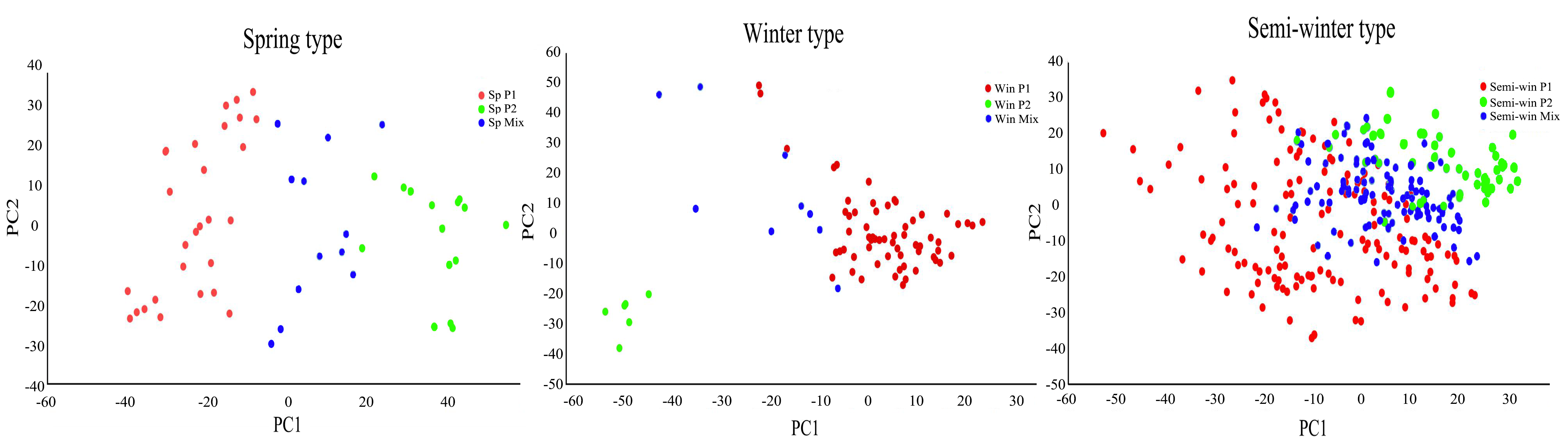

Supplement: Supplementary Figure S1 — Subpopulations in the spring, winter, and semi-winter B. napus accessions, determined using a principal coordinate analysis (PCA). [file Image_1.TIF]
